# A quasi-experimental research on developing students’ life skills through purposeful leisure time activities

**DOI:** 10.3389/fpsyg.2025.1634943

**Published:** 2025-10-15

**Authors:** Göknil Nur Koçak, Mehmet Çağrı Çetin, Mehmet Kara, Murat Genç, Gültekin Lekesiz, Ferhat Caner Açıkbaş, Nuriye Şeyma Kara

**Affiliations:** ^1^Faculty of Tourism, Mersin University, Mersin, Türkiye; ^2^Faculty of Sport Science, Mersin University, Mersin, Türkiye; ^3^Faculty of Sport Science, Hatay Mustafa Kemal University, Antakya, Türkiye

**Keywords:** emotional coping, decision making, empathy and self-awareness, critical and creative thinking, interpersonal communication

## Abstract

This study investigated the efficacy of a camp-based intervention in enhancing students’ life skills and subjective wellbeing. This research examined how student-centered and interactive learning models, implemented through camp activities, contributed to the development of participants’ life skills. Using a quasi-experimental methodology with a pretest/post-test control group design (2 × 2 factorial design), the study employed experimental and control conditions to establish validity. Life skills and subjective wellbeing data were collected through face-to-face administration at consistent time points, before and after the intervention. The study involved 46 students assigned to the experimental group and 42 students assigned to the control group, all of whom were enrolled in the Faculty of Sports Sciences. Statistical analysis employed a mixed-design two-way ANOVA to assess between-group differences and temporal changes, while predictive relationships were examined through correlation and regression analyses. The results demonstrated statistically significant positive changes in both life skills and subjective wellbeing measures among experimental participants compared to their control group counterparts who did not participate in the camp intervention. This finding supports the idea that organized camps can serve as catalysts for improving subjective wellbeing and the development of life skills. As anticipated, enhanced life skill development corresponded to increased subjective wellbeing. The study further determined that interactive structures implemented within the camp framework effectively supported both life skills development and subjective wellbeing enhancement. Camp-based interventions may be recommended as effective tools for developing competencies within organizational human resources, including stress management, problem solving, interpersonal communication, decision-making, and creative and critical thinking.

## 1 Introduction

In contemporary society, individuals consistently encounter challenges in establishing their identities and securing positions within social structures. This developmental process requires the acquisition of specific competencies through formal educational pathways and broader social contexts. These competencies, conceptualized as skills, are defined as the capacity to execute tasks effectively and efficiently, representing abilities acquired or developed through training, practice, and experience (Healy et al., 2014). Skills are fundamental components of personal and professional development that facilitate adaptation to emerging challenges, performance enhancement, and goal attainment (Pintrich and Zusho, 2002). Skills acquired through an individual’s developmental trajectory can be categorized into two primary classifications: hard and soft. Hard skills encompass quantifiable and measurable abilities, including technical knowledge and proficiency, with specific instrumentation or technological applications (Setiana et al., 2019). Soft skills, such as teamwork and problem solving, are characterized by greater subjectivity and typically manifest in interactive or dynamic contextual roles (Hagemann and Kluge, 2017). Together, these classifications constitute ‘life skills’ that fundamentally influence an individual’s capacity to navigate diverse life domains.

Life skills encompass a broad spectrum of behaviors, including communication and decision making, enabling individuals to adapt effectively to various situations and cope with challenges (Trottier and Robitaille, 2014). Life skills instruction enhances individuals’ interpersonal competencies related to relationship formation (Robison et al., 2020) while developing problem-solving capabilities (Lee et al., 2017). The ability to transfer life skills across multiple domains represents a significant additional benefit to their acquisition.

Recent research examining the transfer of life skills from sports contexts to academic environments has garnered substantial scholarly attention. Interventions targeting life skill development and methodologies that facilitate their transfer into educational settings demonstrate considerable potential for enhancing students’ holistic development and academic achievement (Allen et al., 2015). Knowledge and competencies acquired through formal educational processes and social experiences constitute the foundation of an individual’s life skills repertoire. However, for this constellation of knowledge and skills to evolve into sustainable lifelong competencies, a developmental trajectory must follow systematic principles. The World Health Organization (WHO, 1997) proposed a classification of life skills, acknowledged to manifest diverse characteristics across various cultures, into five core sets deemed essential for physically and mentally healthy individuals. The competencies essential for individuals to maintain physical and mental wellbeing can be categorized into five fundamental sets: (1) skills for managing emotions and stress; (2) empathy and self-awareness skills; (3) decision-making and problem-solving skills; (4) creative and critical thinking skills; and (5) communication and interpersonal relationship skills. The cultivation of these competencies is a primary objective of contemporary education.

Emotion regulation and stress-coping skills are essential for individuals as they navigate complex emotional responses in various life contexts. Emotion regulation, defined as the capacity to influence and manage one’s emotional experiences, encompasses both antecedent-focused strategies, such as modifying a situation before an emotion fully develops, and response-focused strategies aimed at modulating the expression or intensity of an emotion after it emerges (Horton et al., 2023; Fitzpatrick, 2018; Zimmermann and Iwanski, 2014). Effective emotion regulation is widely associated with positive mental health outcomes, adaptive psychosocial development, and enhanced academic achievement among university students (Xie et al., 2021; Al-Badareen, 2016).

Despite this, there is a paucity of research on the enhancement of emotional competencies through structured leisure-time interventions. Purposeful camp-based programs may present a promising pedagogical alternative by enabling students to engage in real-world emotionally challenging scenarios where such skills can be actively practiced and reinforced. Therefore, this study aims to address this research gap by examining the impact of experiential, camp-based leisure activities on the development of emotion regulation and stress-coping skills.

Recognizing the developmental importance of these life skills, this study’s decision to adopt a camp-based experiential learning approach was rooted in experiential education theory and bolstered by recent empirical findings. Programs conducted outdoors and in camp settings have been proven to offer enriched environments in which students participate in teamwork, tackle real-time challenges, and reflect on their personal development. Wilson and Sibthorp (2018) illustrated that summer camp settings foster not only emotional resilience and interpersonal skills but also readiness for academic and professional environments. Similarly, James and Williams (2017) discovered that outdoor education within schools enhances students’ decision-making and self-regulation abilities. More recently, Dorland (2024) demonstrated that experiential and design-thinking practices significantly boost creativity, innovation, and collaboration skills in undergraduate classrooms. These studies highlight the relevance and timeliness of utilizing immersive experiential environments to cultivate life skills and psychological competencies.

Empathy and self-awareness are central to students’ social and emotional learning. Self-awareness refers to the capacity to accurately perceive one’s emotions, thoughts, and behaviors and to understand how these internal states influence decisions and relationships (Chance et al., 2023; Rani et al., 2021). Its development has been linked to improved decision making, critical thinking, and interpersonal effectiveness (Hullinger et al., 2019). Empathy, the capacity to understand and share the feelings of others, serves as a foundational component of emotional intelligence, and is critical for the development of meaningful relationships and community engagement (Chance et al., 2023). These abilities are not only emphasized within the Social and Emotional Learning (SEL) framework but are also viewed as pillars of holistic student development (Siraj et al., 2013).

Moreover, the ability to make decisions and solve problems is an essential cognitive skill crucial for achieving success in both academic and professional realms. These metacognitive skills allow individuals to evaluate complex scenarios, consider different options, and make informed decisions. Research has demonstrated that decision-making and problem-solving skills can be greatly enhanced through specific educational programs aimed at various groups (Ahmady and Shahbazi, 2020; Firdaus et al., 2020; Thabet et al., 2017; Heidari and Shahbazi, 2016; Hassan and Mouganie, 2014; Seal, 2006; Güçray, 2005).

Furthermore, both creative and critical thinking are vital to nurturing innovation and intellectual development. Creative thinking promotes the generation of new ideas and effective problem solving through adaptable thought processes, whereas critical thinking emphasizes logical analysis, evidence evaluation, and sound judgment (Fernando et al., 2021; Komaria and Wicaksono, 2019; Romero et al., 2017; Alghafri and Ismail, 2014). The interaction between these cognitive abilities has been demonstrated to enhance deeper learning and to prepare students with the skills needed for future societal contributions (Nafiah et al., 2023; Yusnaeni, 2020). Given its inherent link to problem-solving skills and academic success (Nasir et al., 2022), critical thinking is crucial for thorough information analysis and informed decision (Novferma and Romundza, 2020).

Interpersonal and communicative abilities are vital. These skills encompass empathy, effective expression, conflict resolution, and teamwork, which are essential for both academic success and lifelong achievement (Moradi et al., 2018; Kiuru et al., 2015; Yahaya and Ramli, 2009). Studies indicate that emotional intelligence serves as a mediator in communication effectiveness, while robust communication skills are linked to better academic performance and development of self-concept.

In addition to these essential life skills, subjective wellbeing is a vital psychological concept that involves how individuals assess and emotionally experience life satisfaction, emotional conditions, and overall quality of life (Baker et al., 2005). Among students, subjective wellbeing is shaped by factors such as academic stress, social connections, emotional abilities, and achievement of personal goals. A wealth of research indicates that improving life skills can have a beneficial effect on wellbeing, both by directly enhancing emotional coping mechanisms and indirectly by improving interpersonal relationships and self-efficacy (Schliep et al., 2021; Saw et al., 2016; Chen and Kee, 2008).

Although there is a wealth of research on the individual impacts of life skills and wellbeing, studies that combine both within an experiential educational context are limited. This research aims to fill this gap by examining the effects of a five-day camp-based experiential learning program on enhancing both the cognitive-behavioural aspects of life skills and students perceived subjective wellbeing. The choice of a camp-based approach is supported by the literature that emphasizes the benefits of immersive, social, and physically engaging settings in promoting personal development, teamwork, and emotional resilience (De Prada et al., 2024; Malete et al., 2022; Ghadiri Khanaposhtani et al., 2018).

The theoretical connection between life skills and subjective wellbeing is rooted in cognitive-emotional models, as outlined by Diener et al. (1999). These models suggest that a person’s life satisfaction and emotional health are significantly affected by their capacity to handle challenges, form relationships, and achieve personal objectives. Within this framework, life skills serve as essential tools for improving psychological wellbeing and adaptive functioning (Bean et al., 2015; Yankey and Biswas, 2012). This theoretical basis underlines the idea that various aspects of life skills are crucial for predicting outcomes related to subjective wellbeing. While cognitive-emotional theory clarifies the link between life skills and wellbeing, other frameworks have been employed to guide intervention design and mechanisms. This study is grounded in experiential learning theory (Kolb, 2014), which posits that individuals acquire knowledge and skills through a cyclical process that comprises thoughtful consideration, hands-on practice, theoretical understanding, and active experimentation. Furthermore, the intervention draws on self-determination theory (Ryan and Deci, 2000), which highlights the psychological needs for self-governance, skills, and a sense of belonging as key factors in personal development and subjective wellbeing. These frameworks influenced both the design and the anticipated outcomes of the intervention. As we delve deeper into this relationship, we expect to find evidence to support the hypothesis that there is a strong link between various life skills and overall wellbeing.

This study, grounded in the theoretical framework and addressing the identified research gap, was directed by the following central research question: To what degree are life skills linked to subjective wellbeing, and is it possible to improve these outcomes through a focused, experiential camp-based intervention?

In order to systematically tackle this question, we developed the following hypotheses:

*H1*: There will be a significant positive relationship between life skills and subjective wellbeing at pre-test.

*H2*: There will be a significant positive relationship between life skills and subjective wellbeing at post-test.

*H3*: The subdimensions of life skills will significantly predict individuals’ levels of subjective wellbeing at post-test.

*H4*: Participants in the experimental (camp) group will demonstrate significantly greater improvements in life skills and subjective wellbeing from pre-test to post-test compared to participants in the control group.

## 2 Method

### 2.1 Research design

**Figure 1:**
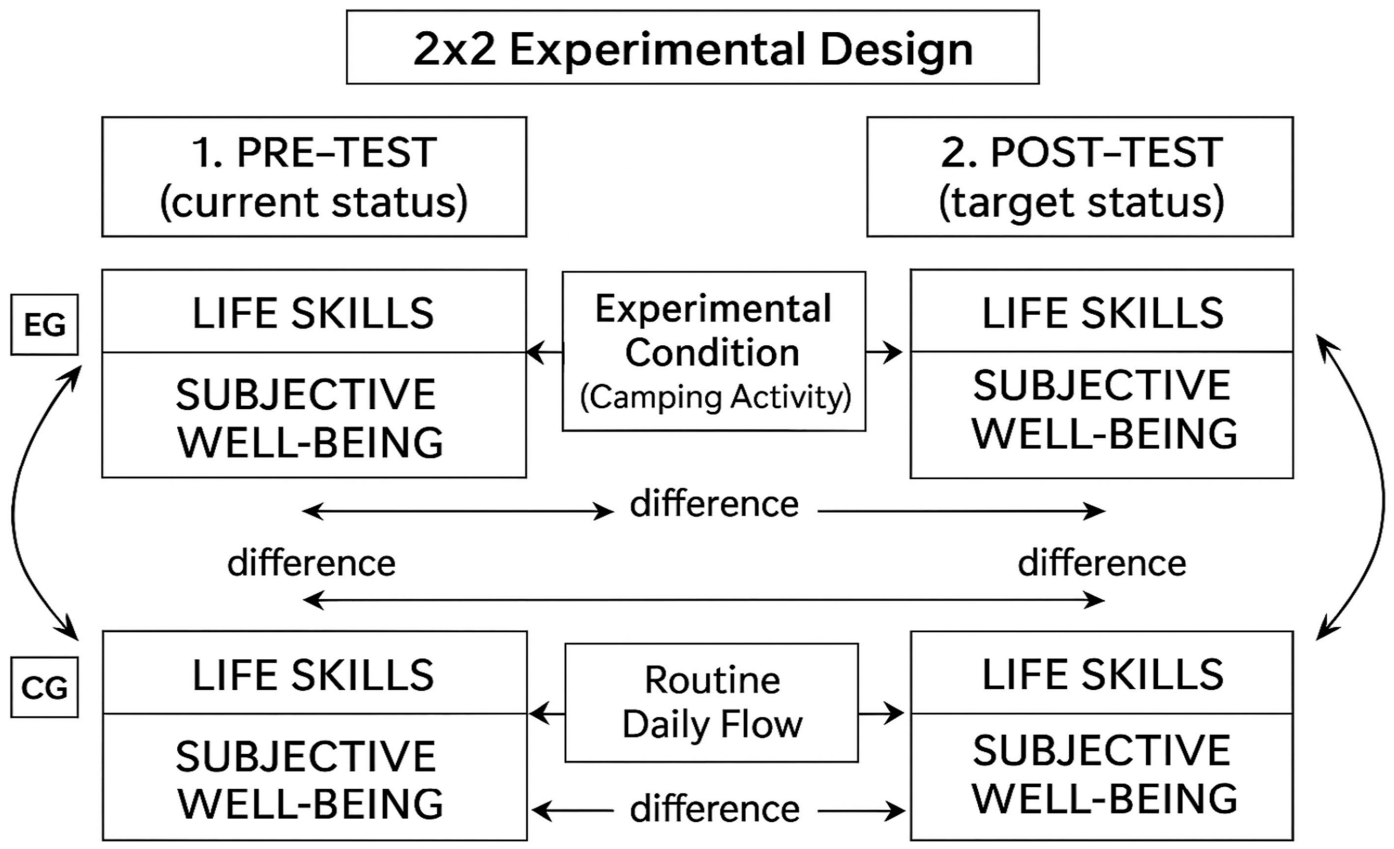
Quasi-experimental research design involving experimental and control group.

The study employed a quasi-experimental design featuring a pretest/post-test control group structure, representing a 2 × 2 factorial layout. Experimental and control groups were incorporated into the design to assess the validity of the findings. One approach to enhance validity involves obtaining data on the same subject through different instruments or methods (Meijer et al., 2002). Employing multiple data sources or methods to corroborate findings is known as ‘triangulation’ (Cao and Reimann, 2020). The inclusion of the experimental and control groups in this study aimed to test the validity of the findings through data triangulation. Both the experimental and control groups were comprised of students from the Faculty of Sports Sciences. Due to the quasi-experimental nature of the study and logistical constraints, the participants were not randomly assigned to groups. Pre-test data for the experimental group were collected via paper-and-pencil instruments administered while the students were traveling en route to the campsite. Control group data were gathered on the same day via face-to-face administration of similar instruments to students who did not participate in the camp and remained in the faculty. Post-test data for the experimental group were collected at the conclusion of the camp during the collective return journey. Post-test data for the control group were collected concurrently with the post-test data collection for the experimental group. A schematic representation of the quasi-experimental research design, illustrating the 2 × 2 factorial structure with pre-test/post-test measures for both the experimental and control groups, is presented in Figure 1.

#### 2.1.1 Techniques implemented for experimental group during the camping activity

In line with the study objectives, a variety of interactive, gamified, competitive, emotionally engaging, innovative, and creativity-enhancing techniques were employed throughout the camping activity. They included improvisation, role-playing and role-switching, hot seating, conscience alley, gossip circle, inner voice, organizing meetings, role cards, thought tracking, map or schema creation, “If I were in your place,” space exploration, a day in the life, and role corridor. The selection of these activities was informed by their potential to positively contribute to students’ innovative and competitive thinking (Richard et al., 2021).

The implemented techniques were designed to ensure active participation of each student, thereby facilitating their full and effective engagement in the process. For example, a role-playing exercise was developed in which students engaged in a simulated conflict over shared resources with the aim of enhancing problem-solving and communication skills. Additional techniques, such as the conscience alley, inner voice, and thought tracking, were employed to improve emotional regulation, perspective-taking, and self-awareness—competencies essential for athletes. Activities such as organizing meetings and utilizing role cards facilitate leadership and team coordination, reflecting the critical components of team sports dynamics. Although these are not sport-specific drills, these experiential methods are consistent with the comprehensive focus of sports psychology on the development of socio-emotional and cognitive skills. In pursuit of this goal, sports faculty students are encouraged to engage in both internal and external observations, allowing for the development of innovative and creative thinking. While the process followed a structured framework, it was conducted with the necessary flexibility to accommodate the natural flow of interpersonal interaction. It is also important to note that camping should not be classified as a traditional natural sport. Natural sports typically include physically demanding and skill-based activities conducted in natural settings, such as hiking, rock-climbing, canoeing, or mountain biking. Conversely, camping is more accurately described as an outdoor leisure activity that entails setting up a temporary shelter or living area in a natural setting, rather than being classified strictly as a sport.

### 2.2 Data collection

#### 2.2.1 Data collection instruments

##### 2.2.1.1 Life skills scale

The Life Skills Scale, developed by Bolat and Balaman (2017), was constructed based on the classification of 10 distinct skill areas into the five core life skills domains previously outlined. All the items within the scale were positively worded. Responses to the skill statements are provided using options ranging from most negative to most positive: ‘Never Agree’, ‘Slightly Agree’, ‘Moderately Agree’, ‘Strongly Agree’, and ‘Completely Agree’. Scoring assigns a value of 5 to the most positive response (‘Completely Agree’) and 1 to the most negative response (‘Never Agree’). Internal consistency reliability coefficients (Cronbach’s alpha) for the sub-factors were calculated as follows: Skills for Coping with Emotions and Stress (*α* = 0.82), Empathy and Self-Awareness Skills (*α* = 0.77), Decision-Making and Problem-Solving Skills (*α* = 0.72), Creative Thinking and Critical Thinking Skills (*α* = 0.73), and Communication and Interpersonal Relationship Skills (*α* = 0.66), which indicated acceptable to good internal consistency for the sub-factors. Cronbach’s alpha coefficient for the entire scale was 0.90, suggesting excellent internal consistency.

##### 2.2.1.2 Subjective wellbeing scale

Developed by Kirmizier (2023), it comprises seven items. A higher total score on this scale indicates a higher level of subjective wellbeing. All items were positively worded. Responses are provided on a 5-point scale ranging from ‘Not at all’ to ‘Very much’. Scores ranged from 1 (‘not at all’) to 5 (‘very much’). The Kaiser-Meyer-Olkin (KMO) measure of sampling adequacy for the scale was 0.797. The scale structure explained 47% of the total variance, and the Cronbach’s alpha internal consistency coefficient was 0.787. Both instruments meet established standards for validity and reliability in measurements. Consistent results were also obtained in the test–retest reliability analyses conducted during the respective scale development phases (Table 1).

**Table 1:** Confirmatory factor analysis fit indices for data collection instruments.

Construct	CMIN/DF (*χ2/df*)	CFI	TLI	SRMR	RMSEA	Cronbach’s *α*
Subjective wellbeing	24.5/14 = 1.75	0.95	0.93	0.044	0.020	0.84
Life Skills	776/395 = 1.96	0.80	0.80	0.077	0.080	0.96

Confirmatory Factor Analysis (CFA) results for the Subjective Wellbeing Scale were as follows: CMIN/DF (χ2/df) = 1.75, CFI = 0.95, TLI = 0.93, SRMR = 0.044, RMSEA = 0.020. The Cronbach’s Alpha (*α*) internal consistency coefficient was 0.84. For the Life Skills Scale, the CFA results were: CMIN/DF (χ2/df) = 1.96, CFI = 0.80, TLI = 0.80, SRMR = 0.077, RMSEA = 0.080. The Cronbach’s Alpha (α) internal consistency coefficient was calculated as 0.96.

Interpreting the CFA results for the Subjective Wellbeing scale, the CMIN/DF (*χ2/df*) value of 1.75 is below the commonly accepted threshold of 5, indicating an acceptable model fit according to this criterion (Kline, 2023). The CFI (0.95) and TLI (0.93) values exceed the 0.90 benchmark, providing evidence of a good model fit (Hu and Bentler, 1999). The SRMR (0.044) and RMSEA (0.020) values suggest excellent model fit. Values below 0.08 for SRMR and below 0.06 RMSEA are typically considered indicative of acceptable fit (Hooper et al., 2008). Cronbach’s alpha (α), indicating internal consistency, was 0.84. Values above 0.70 are generally considered to indicate adequate reliability (Nunnally and Bernstein, 1994). Therefore, the Subjective Wellbeing Scale demonstrates construct validity and reliability that meet established criteria.

The CFA for the Life Skills Scale yielded an acceptable chi-square to degrees of freedom ratio (*χ^2^/df* = 1.96), aligning with the established standards (Kline, 2023). Both CFI and TLI values reached 0.80, and while these fall short of the optimal 0.90 threshold, they met the minimum acceptable criteria for adequate model fit, as suggested in the literature (Marsh et al., 2004). The SRMR (0.077) remained below the conventional 0.08 cutoff, demonstrating an acceptable fit. Similarly, RMSEA (0.080) stood at the upper boundary of what researchers typically considered reasonable (Browne and Cudeck, 1992). The scale exhibited exceptional internal consistency, with a Cronbach’s alpha coefficient of 0.96, reflecting superior reliability according to established interpretive guidelines (George and Mallery, 2003).

### 2.3 Sample description

The adequacy of the sample size was assessed through a post-hoc power analysis using G*Power 3.1 software (Faul et al., 2007). This analysis was tailored to the study’s primary hypothesis test, specifically the 2 × 2 within-between interaction from the mixed-design ANOVA. Based on the observed effect size (partial *η^2^* = 0.087, corresponding to *f* = 0.31), a value considered to be a medium-to-large effect (Cohen, 2013), the achieved power (1-*β*) for the total sample of *N =* 88 (comprising two groups and two measurements at an alpha level of 0.05) was 0.82. This value surpasses the conventional threshold of 0.80, indicating that the study was sufficiently powered to detect an effect of the observed magnitude.

### 2.4 Ethical considerations

This study was conducted in accordance with the Declaration of Helsinki and approved by the Institutional Review Board of Mersin University (29/07/2024–48).

### 2.5 Data collection procedure

In this study, data were collected at two distinct time points, pre-camp and post-camp, to examine the effects of the camping intervention. A paper-and-pencil format was utilized for data collection to allow participants to reflect on and record their thoughts and assessments. Pre-camp measurements were conducted on the first day before the camp commenced and involved the individual distribution of data collection forms to the participants. During this session, the participants completed the Subjective Wellbeing Scale and Life Skills Scale. Throughout the camp duration, the experimental group participated in planned activities and various training programs. During the intervention period, the control group did not participate in any alternative structured activities and continued their regular academic routines. Following the camp period (post-camp), the same measurement instruments (Wellbeing Scale and life skills scales) were re-administered to the control group participants. This procedure established a pre-test/post-test design for both camp attendees (experimental group) and non-attendees (control group), facilitating the observation of changes over the camp period.

Appropriate arrangements were made within the data collection settings to ensure that the participants could complete the forms comfortably. Data collection was based on voluntary participation, and all information obtained from the participants was kept confidential. Upon completion of the data collection phases, comparative analyses were conducted between the experimental and control groups to examine the effectiveness of the camp intervention in detail. This procedure enabled a comparative analysis of changes within the experimental group relative to the control group. This comparison enhanced the reliability of the study’s conclusions regarding the effects of the intervention.

### 2.6 Data analysis

To satisfy the requirements of a two-way mixed-design ANOVA (2 × 2), the assumption of normal distribution must be met across all groups and measurements within the analysis. This assumption is verified when Kolmogorov–Smirnov (K-S) test results yield *p*-values exceeding 0.05 (Greenland et al., 2016). As shown in Table 2, all distributions demonstrated p-values above this threshold, confirming normality across all subgroups and measurements. With normality established, we proceeded with the two-way mixed ANOVA to evaluate the camp program’s impact on the experimental group. The analytical framework employed a two-way design, incorporating two independent variables: group (experimental versus control) and time (pre-test versus post-test). This methodological approach facilitates concurrent examination of between-group variations, within-group temporal changes, and their interaction effects (Jansen et al., 2005). The two-way mixed ANOVA methodology enables comprehensive assessment of group differences, longitudinal changes, and group-by-time interactions while controlling error rates and maintaining statistical precision within a 95% confidence interval (Fidler and Thompson, 2001). In addition to ANOVA, we employed simple linear regression analysis to investigate the relationship between life skills and subjective wellbeing. Statistical analyses were conducted utilizing IBM SPSS Statistics for Windows, Version 25.0 (IBM Corp., Armonk, N.Y., USA) (Table 3).

**Table 2:** Normality test for ANOVA.

			*M*	*n*	K-S Statistic	*p*
Pre-test	Subjective Wellbeing	Experimental	4.20	46	0.126	0.064
		Control	3.80	42	0.103	0.200
	Life Skills	Experimental	4.17	46	0.129	0.053
		Control	3.92	42	0.084	0.200
Post-test	Subjective Wellbeing	Experimental	4.44	46	0.159	0.055
		Control	3.72	42	0.125	0.099
	Life skills	Experimental	4.48	46	0.186	0.060
		Control	3.87	42	0.085	0.200

*Significant at *p* < 0.05.

**Table 3:** Mixed-design ANOVA results between groups.

Source of variation	Sum of squares	df	Mean square	*F*	*p*
Within-subjects effect					
Time (Factor 1)	0.814	1	0.814	4.690	0.033*
Time * Group (Factor 2)	1.420	1	1.420	8.185	0.005**
Error	14.919	86	0.173		
Between-subjects effect					
Intercept	2974.098	1	2974.098	7493.139	0.00***
Group	8.151	1	8.151	20.537	0.00***
Error	34.134	86	0.397		

*Significant at *p* < 0.05, **Significant at *p* < 0.01, and *** Significant at *p* < 0.001.

## 3 Results

Statistical analyses were performed to assess the effects of the camp intervention on students’ life skills and subjective wellbeing. The findings are presented in order, starting with the primary ANOVA, followed by correlational and regression analyses.

To test our primary experimental hypothesis (H4), a two-way mixed-design ANOVA was utilized to investigate the intervention’s impact. The analysis indicated a statistically significant main effect for time, *F*_(1, 86)_ = 4.690, *p* = 0.033, and a significant interaction effect between Time and Group, *F*_(1, 86)_ = 8.185, *p* = 0.005. Additionally, a significant main effect for group was identified, *F*_(1, 86)_ = 20.537, *p* < 0.001.

**Figure 2:**
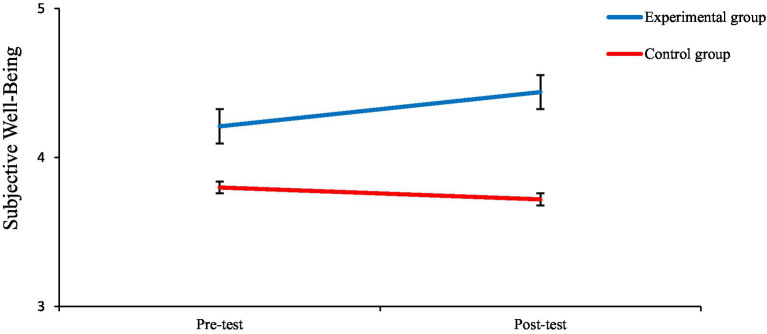
Subjective wellbeing scores for experimental and control groups in pre- and post-test sessions. The experimental group demonstrated a clear increase in subjective wellbeing from pre- to post-test, whereas the control group showed a slight decline. Error bars represent ±1 SE.

**Figure 3:**
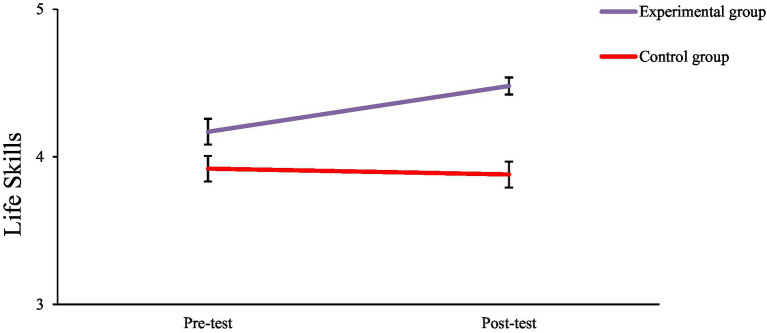
Life skills scores for experimental and control groups in pre- and post-test sessions. The experimental group showed a marked improvement in life skills, while the control group’s scores remained relatively stable across time. Error bars represent ±1 SE.

Analysis of the estimated marginal means, as illustrated in Figures 2, 3, confirms the presence of an interaction effect. Regarding subjective wellbeing, the mean score for the experimental group increased from 4.20 before the test to 4.44 afterward, whereas the mean score of the control group exhibited minimal change, shifting from 3.80 3.72. Similarly, in terms of life skills, the experimental group’s mean rose from 4.17 to 4.48, while the control group’s mean remained nearly constant, moving from 3.92 to 3.87.

Two-way mixed-design ANOVA was used to examine the effects of the intervention. The results revealed a statistically significant main effect for time, *F*_(1, 86)_ = 4.690, *p* = 0.033, and a significant main effect for group, *F*_(1, 86)_ = 20.537, *p* < 0.001. Importantly, there was a significant interaction effect between Time and Group, *F*_(1, 86)_ = 8.185, *p* = 0.005. This significant interaction suggests that the change from pre-to post-test differed between the two groups. As shown in Figures 2, 3, the experimental group experienced a noticeable increase in the mean scores for both subjective wellbeing (4.20 to 4.44) and life skills (from 4.17 to 4.48). Conversely, the control group’s mean scores remained relatively unchanged for life skills (3.92 to 3.87) and decreased for subjective wellbeing (3.80 to 3.72). The statistically significant interaction effect (*p* = 0.005) confirms that this observed difference in trends between the groups is not attributable to chance.

As shown in Table 4, Pearson correlation analyses were performed on the post-test data. The findings indicated statistically significant, moderate, positive correlations between subjective wellbeing and each sub-dimension of the Life Skills Scale. Additionally, there were strong positive intercorrelations among all life skills sub-dimensions, with the strongest connection identified between Decision-Making and Problem-Solving Skills and Creative Thinking and Critical Thinking Skills (*r* = 0.82).

**Table 4:** Pearson correlation matrix for post-test variables of scales.

Variables	*M*	*SD*	1	2	3	4	5	6
1. Subjective wellbeing	3.97	0.656	1	0.33**	0.21**	0.32**	0.28**	0.36**
2. Skills for coping with emotions and stress	3.77	0.693		1	0.61**	0.60**	0.59**	0.50**
3. Empathy and self-awareness skills	4.13	0.658			1	0.76**	0.74**	0.69**
4. Decision-making and problem-solving skills	4.09	0.658				1	0.82**	0.74**
5. Creative thinking and critical thinking skills	4.18	0.764					1	0.73**
6. Communication and interpersonal relationship skills	4.09	796						1

**Correlation is significant at the *p* < 0.01 level. *Correlation is significant at the *p* < 0.05 level.

The following regression equation (mathematical model) is derived from the regression analysis results to forecast Subjective Wellbeing:

Subjective Well-being^1^ = 2.548 + (0.245 × coping) + (−0.254 × empathy) + (0.160 × Decision) + (−0.540 × creative) + (0.275 × communication).

Multiple linear regression analysis was conducted to evaluate how well the five dimensions of life skills could predict subjective wellbeing. The subdimensions of the Life Skills Scale served as independent variables, while subjective wellbeing was the dependent variable. The analysis met all assumptions, indicating no issues with multicollinearity (all tolerances > 0.25, VIF < 4.0) or independence of errors (Durbin-Watson = 1.631). The overall regression model was statistically significant, *F*(5, 82) = 4.295, *p* = 0.001, explaining 18.6% of the variance in subjective wellbeing (Adjusted *R*^2^ = 0.143). A closer look at the individual predictors (Table 5) revealed that coping skills with emotions and stress (*β* = 0.259, *p* = 0.038) and Communication and Interpersonal Relationship Skills (*β* = 0.334, *p* = 0.025) were significant positive predictors. The other three dimensions— empathy and self-awareness skills, decision-making and problem-solving skills, and creative and critical thinking skills —did not reach statistical significance in the model.

**Table 5:** Multiple linear regression analysis.

Model 1	*B*	*SE*	*(β)*	*T*	*p*	Tolerance VIF	
Constant	2.548	0.424		6.00	0.000		
1. Skills for coping with emotions and stress	0.245	0.116	0.259	2.107	0.038	0.574	1.742
2. Empathy and self-awareness skills	−0.254	0.159	−0.253	−1.597	0.114	0.346	2.887
3. Decision-making and problem-solving skills	0.160	0.183	0.160	0.871	0.386	0.256	3.913
4. Creative thinking and critical thinking skills	−0.054	0.154	−0.063	−0.350	0.727	0.269	3.724
5. Communication and interpersonal relationship skills	0.275	0.121	0.334	2.272	0.025	0.401	2.492

Dependent Variable: Subjective wellbeing. *p* = 0.001*F(5, 82) = 4.295, D-W = 1.631, *R* = 0.431, R2 = 0.186, Adj. R2 = 0.143. **Correlation is significant at the *p* < 0.01 level. *Correlation is significant at the *p* < 0.05 level.

## 4 Discussion, conclusion, and recommendations

### 4.1 Discussion of main findings

The findings of this study offer robust support for the research hypotheses. As anticipated by H1 and H2, significant positive correlations were identified between life skills and subjective wellbeing. H3 received partial support, as the regression analysis revealed that Skills for Coping with Emotions and Stress, along with Communication and Interpersonal Relationship Skills, are significant predictors of wellbeing. Notably, H4 was fully supported, as the significant Time × Group interaction from the ANOVA confirmed the intervention’s effectiveness. Specifically, the student-centered camp program resulted in statistically significant enhancements in both life skills and subjective wellbeing for the experimental group, whereas no such changes were observed in the control group. This outcome aligns with an expanding body of research indicating that experiential learning environments are highly effective for socio-emotional development (De Prada et al., 2024; Ghadiri Khanaposhtani et al., 2018).

The improvements observed across all life skills domains emphasize the cross-contextual applicability of skills acquired in an immersive setting, supporting previous research on life skills transfer (Trottier and Robitaille, 2014). The specific predictive power of stress-coping and communication skills is particularly significant. This implies that the interactive, collaborative, and often unpredictable nature of camp activities offered a strong context for participants to practice and enhance these specific skills. This aligns with foundational wellbeing theories that emphasize emotional regulation and strong social connections as key components of life satisfaction (Diener et al., 1999). The consistent pattern of results from the statistical analyses suggests that experiential learning methods and collaborative team-building exercises used in the camp curriculum were crucial in facilitating these positive developmental changes.

These findings are consistent with the theoretical models that informed the research. The experiential learning theory (Kolb, 2014) highlights the importance of learning through doing, reflecting, and experimenting, which is reflected in the design of camp activities. Similarly, the autonomy-supportive setting aligns with the self-determination theory, which fosters intrinsic motivation and psychological health by emphasizing competence, autonomy, and social connections. These theories help clarify why even a short intervention could have a significant short-term impact on participants’ development.

### 4.2 Limitations and directions for future research

Although the findings of this study offer valuable insights, it is essential to interpret them within the context of the research design. The quasi-experimental approach was selected because of its high ecological validity in a real-world field setting; however, this choice implies that participants were not randomly assigned to groups. Future research employing a randomized controlled trial, where feasible, could further enhance causal inferences drawn from such interventions.

Additionally, the study examined the immediate effects of a short-term, five-day intervention. Although the significant changes observed are promising, an intriguing avenue for future research is to conduct longitudinal follow-up assessments. Such studies would enable an examination of the long-term persistence of skills and wellbeing enhancements, providing a more comprehensive understanding of the intervention’s lasting impact. Adding booster sessions or incorporating experiential elements into formal curricula could also help to maintain progress over time.

The current study relied on self-report measures, which are standard and effective methods for assessing subjective constructs such as wellbeing and perceived skills. To build on these findings, future research could incorporate multi-method approaches, including observational data or peer assessments, to provide a more triangulated perspective on participant development.

Finally, our regression model identified key predictors of subjective wellbeing but also indicated that other factors played a role. This presents opportunities for future studies to explore additional personal and contextual variables that may contribute to students’ wellbeing in experiential learning settings.

### 4.3 Conclusion and recommendations

In summary, this research offers important quasi-experimental evidence that a brief, intensive camp program can greatly improve university students’ life skills and subjective wellbeing. These results underscore the critical role of developing stress management and communication skills as essential elements of wellbeing. This study adds to the existing literature by showing the practical benefits of experiential learning in a nonformal educational context. It also emphasizes fundamental psychological mechanisms, such as independence, introspection, and interaction with peers, that facilitate these developmental improvements.

Based on these results, the following suggestions are made:

Universities and other educational bodies should consider incorporating structured, participant-focused experiential learning methods into their curricula to promote students’ overall development beyond conventional academic learning. The principles of this intervention can also be adapted for use in corporate and organizational environments to boost workforce skills (Zhang et al., 2019).

Program Design: We suggest creating camp programs with modular flexibility to cater to the unique needs of individual participants. Offering a variety of skill-specific activities aimed at different life skills may support the realization of individual potential (Amran et al., 2019).

## Data Availability

The raw data supporting the conclusions of this article will be made available by the authors, without undue reservation.
